# Ensemble Deep Learning Models for Automated Segmentation of Tumor and Lymph Node Volumes in Head and Neck Cancer Using Pre- and Mid-Treatment MRI: Application of Auto3DSeg and SegResNet

**DOI:** 10.1007/978-3-031-83274-1_21

**Published:** 2025-03-03

**Authors:** Dominic LaBella

**Affiliations:** Department of Radiation Oncology, Duke University Medical Center, Durham, NC 27710, USA

**Keywords:** Artificial Intelligence, Automated Segmentation, Deep learning, Auto3DSeg, MONAI, Head and neck tumors, Radiation Oncology

## Abstract

Automated segmentation of gross tumor volumes (GTVp) and lymph nodes (GTVn) in head and neck cancer using MRI presents a critical challenge with significant potential to enhance radiation oncology workflows. In this study, we developed a deep learning pipeline based on the SegResNet architecture, integrated into the Auto3DSeg framework, to achieve fully-automated segmentation on pre-treatment (pre-RT) and mid-treatment (mid-RT) MRI scans as part of the DLaBella29 team submission to the HNTS-MRG 2024 challenge. For Task 1, we used an ensemble of six SegResNet models with predictions fused via weighted majority voting. The models were pre-trained on both pre-RT and mid-RT image-mask pairs, then fine-tuned on pre-RT data, without any pre-processing. For Task 2, an ensemble of five SegResNet models was employed, with predictions fused using majority voting. Pre-processing for Task 2 involved setting all voxels more than 1 cm from the registered pre-RT masks to background (value 0), followed by applying a bounding box to the image. Post-processing for both tasks included removing tumor predictions smaller than 175–200 mm^3^ and node predictions under 50–60 mm^3^. Our models achieved testing DSCagg scores of 0.72 and 0.82 for GTVn and GTVp in Task 1 (pre-RT MRI) and testing DSCagg scores of 0.81 and 0.49 for GTVn and GTVp in Task 2 (mid-RT MRI). This study underscores the feasibility and promise of deep learning-based auto-segmentation for improving clinical workflows in radiation oncology, particularly in adaptive radiotherapy. Future efforts will focus on refining mid-RT segmentation performance and further investigating the clinical implications of automated tumor delineation.

## 1 Introduction

The 2024 Head and Neck Tumor Segmentation (HNTS-MRG 24) challenge aims to advance the precision of oropharyngeal cancer (OPC) treatment by improving gross tumor volume (GTV) delineation through novel imaging techniques, particularly focusing on MRI T2 sequences in both pre-radiotherapy and mid-radiotherapy settings. OPC, a subtype of head and neck squamous cell carcinoma, continues to affect a significant population worldwide [1]. Accurate tumor segmentation is vital for delivering effective radiation therapy, as it ensures an optimal therapeutic dose to the tumor while sparing surrounding healthy tissues [2]. Traditionally, segmentation relied on computed tomography (CT) and positron emission tomography (PET) to provide both anatomical and functional information, but these modalities often face limitations due to inter-observer variability and inconsistency in tumor delineation [3, 4]. To address this, MRI T2 has emerged as a powerful modality, particularly for head and neck cancers, as it offers superior soft tissue contrast, which is essential for more precise tumor visualization and improved treatment planning [5]. MRI T2 is particularly useful in the context of OPC, where accurate identification of tumor boundaries in pre-radiotherapy and mid-radiotherapy stages can significantly impact treatment outcomes [6, 7]. Leveraging MRI T2 in these critical phases can enhance the accuracy of tumor tracking and reduce variability in segmentation, thus improving radiation targeting, especially in the adaptive radiotherapy planning setting [8].

This work presents the results of our OPC auto-segmentation model utilizing MRI T2 sequences, focusing on the pre-radiotherapy and mid-radiotherapy setting, predicting both gross tumor and nodal volumes, as part of the 2024 HNTS-MRG challenge.

## 2 Methods

We utilized a deep learning architecture, Auto3DSeg, in conjunction with the MONAI framework to develop auto-segmentation models for oropharyngeal cancer (OPC) patients [9, 10]. For Task 1, the model was trained to predict tumor and node volumes using pre-radiotherapy MRI T2 images for segmentation. Task 2 extended this approach by incorporating both registered-pre-radiotherapy and mid-radiotherapy MRI T2 images with the registered-pre-radiotherapy ground truth GTVp and GTVn labels to predict GTVp and GTVn volumes at the mid-radiotherapy stage.

### 2.1 Imaging Data

The dataset used in this study was released through the HNTS-MRG 2024 challenge at MICCAI 2024, as available on Zenodo [11]. This dataset includes MRI scans and corresponding segmentation masks for patients with head and neck cancer, primarily oropharyngeal cancer, who underwent radiotherapy at The University of Texas MD Anderson Cancer Center. The dataset consists of pre-radiotherapy T2-weighted MRI scans taken 1–3 weeks before the start of radiotherapy and mid-radiotherapy T2-weighted MRI scans taken 2–4 weeks into treatment. For each patient, segmentation masks for the gross tumor volumes and involved lymph nodes are provided, derived from multi-observer STAPLE consensus.

The HNTS-MRG 2024 challenge is divided into two tasks: Task 1 involves segmenting tumor and nodal volumes using pre-RT MRI, while Task 2 requires segmentation on mid-RT MRI using the registered-pre-radiotherapy image, mid-radiotherapy image, and the registered-pre-radiotherapy ground GTVp and GTVn masks. A single training dataset containing 150 unique patients has been provided for both tasks. During the testing phase, different test data will be used for each task and this data is private to the organizers.

All imaging data, including segmentation masks, are provided in Neuroimaging Informatics Technology Initiative (NIfTI) format to facilitate ease of use. Pre-RT and mid-RT MRI scans are available in either fat-suppressed or non-fat-suppressed versions, with no exogenous contrast agents used. The dataset also includes registered pre-RT images and segmentation masks aligned to the mid-RT image space, generated using SimpleITK with both rigid and deformable transformations for ease of comparison in real-world adaptive radiotherapy settings [12–14].

The dataset is structured in a standardized format and contains anonymized patient identifiers. Pre-RT and mid-RT data are organized by patient ID and timepoint, with consistent labeling conventions across cases.

In order to develop effective pre- and post-processing techniques for challenge test set evaluation for both Task 1 and Task 2, analysis of the training sets GTVp and GTVn instance-lesion mask volumes was performed. Specifically, radial shrinkage was performed at varying integer amplitudes from 0–5 mm for the registered-pre-radiotherapy instance-lesion mask volumes for comparison to the respective mid-radiotherapy instance-lesion mask volumes. Analysis of the GTVp and GTVn instance-lesion mask volumes for the entire 150 cases training set was performed and the associated analysis code is available at (github.com/dlabella29/HNTSMRG_2024_DL_Public). The code to perform the inner margin radial shrinkage is available under the erode_mask method of the calcDiceMarginsTask2allPreRT.py script.

### 2.2 Image Pre-Processing

#### Task 1

**Fig. 1.**
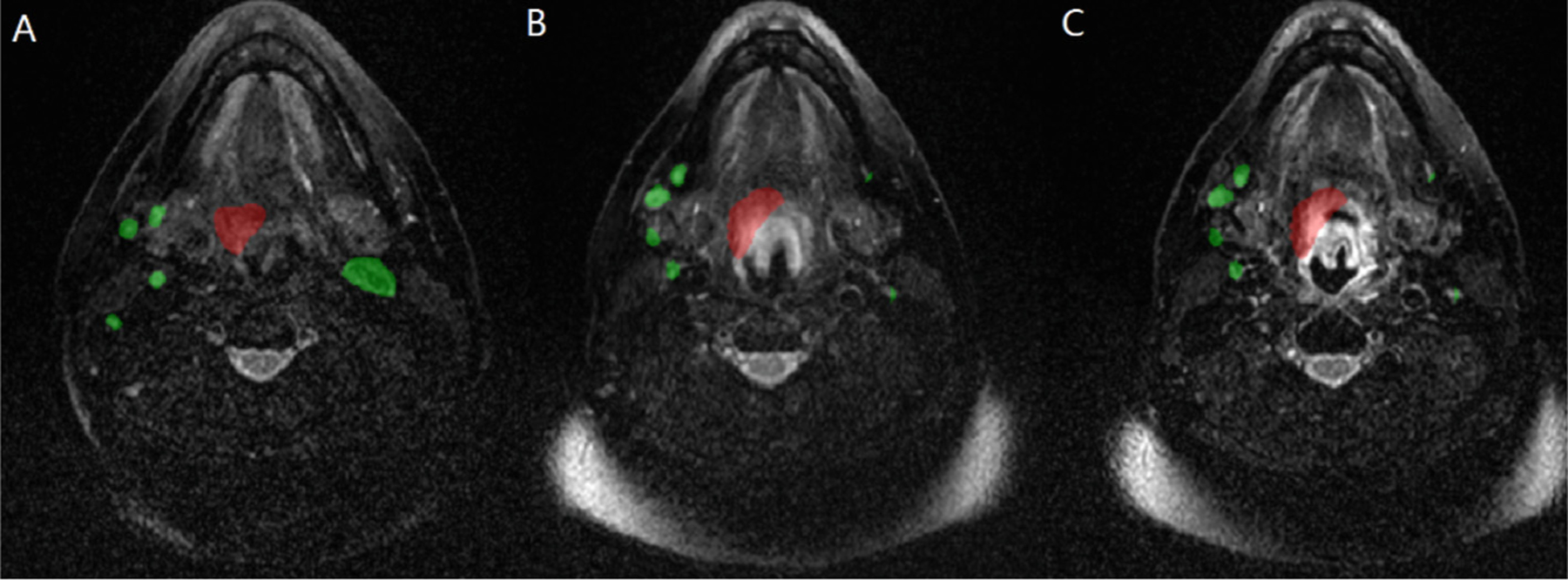
An illustration of the unmodified axial T2 MRI of case number 155 with tumor label (red) and nodal labels (green) for the (A) pre-radiotherapy, (B) registered pre-radiotherapy, and (C) mid-radiotherapy image-mask pairs. (Color figure online)

There was no pre-processing performed for Task 1 of any image or mask data. All of the unmodified pre-radiotherapy, registered-pre-radiotherapy, and mid-radiotherapy image-mask pairs were used in an initial pre-trained model as shown in Fig. 1. Only the unmodified pre-radiotherapy image-mask pairs were included in the fine-tuned models as shown in Fig. 1A.

#### Task 2

**Fig. 2.**
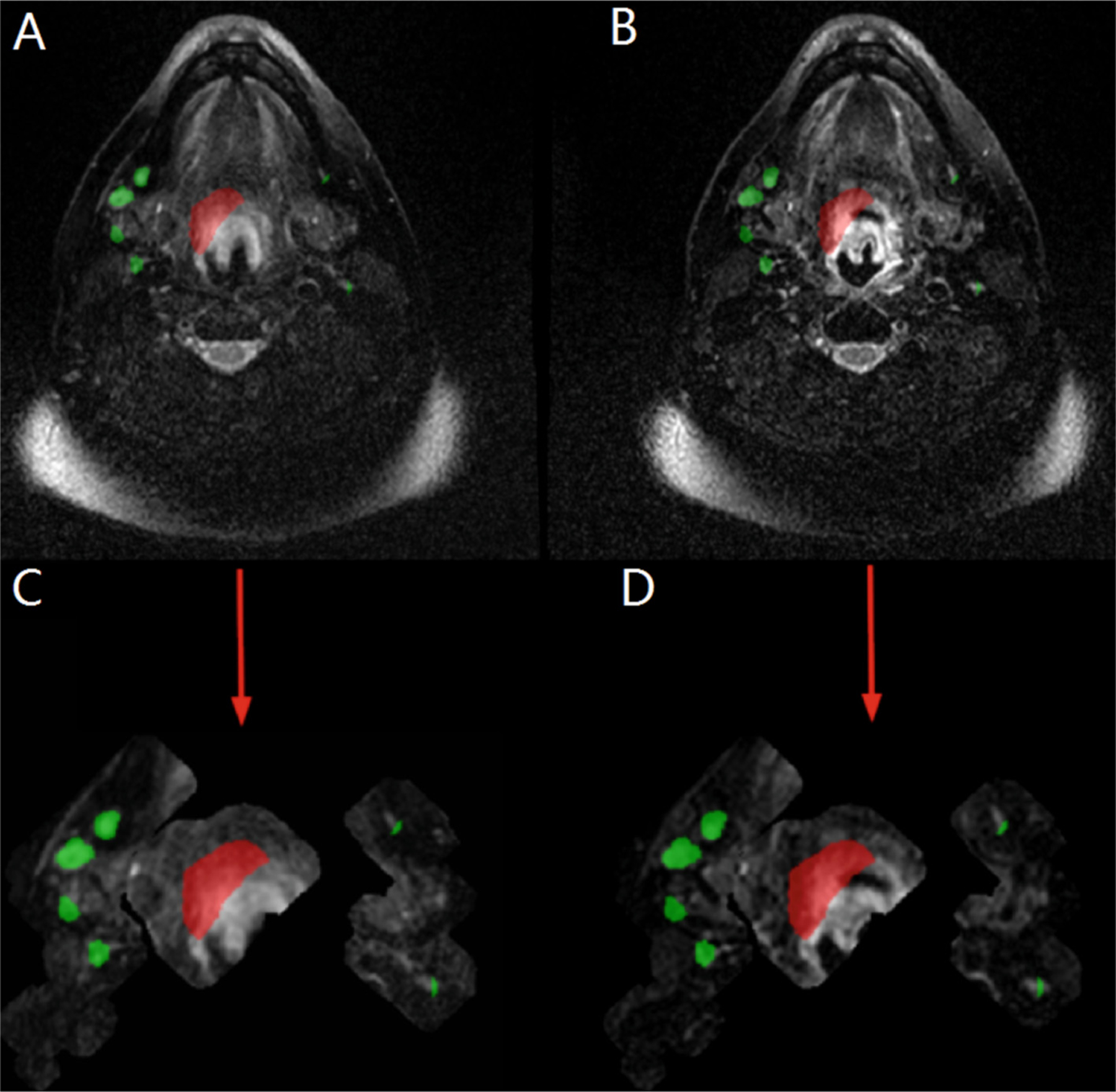
An illustration of the axial T2 MRI of case number 155 demonstrating pre-processing completed for the Task 2 training dataset which included initial (A) registered-pre-radiotherapy T2 MRI and (B) mid-radiotherapy T2 MRI that were reduced in size and foreground cropping to 1 cm radially around all GTVp (red) and GTVn (green) labels as shown in (C) and (D). (Color figure online)

Task 2 pre-processing included modification of both the registered-pre-radiotherapy and the mid-radiotherapy T2 MRI, which are shown in Figs. 2A and 2B. The modifications were performed by radially expanding the GTVp and GTVn labels by 1 cm. This expanded region was then used as a foreground for cropping of the original registered-pre-radiotherapy and the mid-radiotherapy T2 MRI. Then, an additional 0.5 cm x, y, and z directional expansion was made on the bounding box of the new cropped foreground to make the final modified registered-pre-radiotherapy and the mid-radiotherapy T2 MRI, which are shown in Figs. 2C and 2D. This pre-processing was performed to allow for a smaller focused area for SegResNet training on the most pertinent areas of GTVp and GTVn involvement. Since the pre-radiotherapy masks are provided as input data for the Task 2 challenge, and since training set anaylsis showed that there was no significant evidence of GTVp or GTVn growth greater than 1 cm between registered-pre-radiotherapy and mid-radiotherapy cases, there was little concern for potential exclusion of GTVp or GTVn disease on the mid-radiotherapy MRI.

### 2.3 Model Architecture and Implementation

We employed a deep learning convolutional neural network model based on the Seg-ResNet architecture, implemented using the MONAI and Auto3DSeg frameworks [9, 10]. The Auto3DSeg framework is an automated medical image segmentation platform designed to streamline the development, training, and inference of 3D segmentation models [9, 10]. It integrates state-of-the-art deep learning algorithms with a modular, pipeline-based architecture to efficiently handle the entire workflow. Auto3DSeg lever-ages advanced techniques such as automated hyperparameter tuning, architecture search, and multi-fold training strategies to optimize model performance for specific datasets [9, 10]. Its flexibility allows for customization while offering pre-defined configurations for various segmentation tasks [9, 10]. The framework supports diverse input formats, multi-class labeling, and domain-specific constraints, making it a robust solution for clinical and research applications [9, 10]. SegResNet is an encoder-decoder based semantic segmentation network, with the initial filters set to 32 [9, 10]. The encoder used 5 ResNet blocks with instance normalization. The downsampling included 5 stages with 1, 2, 2, 4, and 4 convolutional blocks, respectively. Data augmentation included random flipping on all axes, random rotation and scaling, random smoothing, noise, intensity scale and shifting. We trained the model using global (patient level or traditional volumetric) Dice scores and focal cross entropy loss. We used an AdamW optimizer with a learning rate of 0.0002 and a weight decay of 0.00001. We used a batch size of 1 and 1 image per batch was used. The MRI T2 images served as input, with each voxel classified as either background, GTVp, or GTVn in the output mask. In Task 1, only the pre-radiotherapy T2 MRI was used as input. In Task 2, the modified registered-pre-radiotherapy and modified mid-radiotherapy T2 MRI were used as input. No further data-augmentation was performed in order to limit the size of the training dataset to speed up training time. All training was conducted on a laptop computer with an RTX 2070 GPU and 16 GB of available RAM.

For Task 1, the model training involved two stages. First, a pre-trained model was developed over 802 epochs, with a planned total of 900 epochs. Pre-training was not completed to all 900 planned epochs due to model crashing during training and limitation on time to allow for re-training. The pre-trained dataset included each of the pre-radiotherapy, mid-radiotherapy, and registered-pre-radiotherapy image-mask pairs, all as separate training cases as shown in Fig. 1. Fine-tuning of this pre-trained model was conducted. The fine-tuning dataset only included the pre-radiotherapy image-mask pairs shown in Fig. 1A, as the pre-radiotherapy MRI are the only images provided as input data in the Task 1 challenge. There was a plan for 556 epochs using cross-validation with a planned 8 folds. The folds were split to equally have 12.5% of the total training set data, with no data left out for independent testing. The Json files for training splits, Python scripts to generate the Json files, and Python scripts to conduct model training are available at (github.com/dlabella29/HNTSMRG_2024_DL_Public). The number of epochs of 556 was predicted by the Auto3DSeg architecture [9, 10]. Unfortunately, due to training time limitations, only 6 of the planned 8 folds underwent fine-tune training. The models for the 7^th^ and 8^th^ folds were not run. Fold 0 utilized the same validation set’s pre-radiotherapy MRI as the pre-trained model’s pre-radiotherapy pre-training dataset, and this fold successfully completed all 556 epochs. Folds 1–5 used new validation set’s pre-radiotherapy images compared to the pre-trained model’s training dataset, but each of these respective validation set’s images were a part of the pre-training datasets training images. Therefore, artificially high validation scores were achieved early on during training during the fine-tuning for folds 1–5 as shown in Table 1. Additionally, not all of the folds 1–5 made it to completion as shown in Table 1. Re-training was not feasible due to time limitations. A weighted majority voting ensembling technique was employed with the model weights shown in Table 1. Fold 1 was assigned the highest weight, since this fine-tuned model used the same validation pre-radiotherapy image-mask pairs as those utilized in the pre-trained model. Folds 1 and 5 had lower relative weights due to the models crashing early during the training process. The best validation scores were achieved early on during each of the folds 0–5 fine-tuning. This is likely due to over-fitting on the features associated with the validation cases that were previously used in the pre-trained model’s training sets. Therefore, each fold’s respective final model was used for inference instead of the best epoch models.

For Task 2, the model training involved a single stage without any pre-training or fine-tuning. A total of 534 epochs were successfully completed for each of the 5 planned folds. The folds were split to equally have 20% of the total training set data, with no data left out for independent testing. Training summaries are provided in Table 2.

For Task 1 and Task 2, all the Python code for pre-processing, training, inference, ensembling, and post-processing are publicly available (github.com/dlabella29/HNTSMRG_2024_DL_Public). The inference code was modified from the Auto3DSeg architecture inference methods (github.com/Project-MONAI/tutorials/tree/main/auto3dseg).

### 2.4 Model Post-Processing

**Fig. 3.**
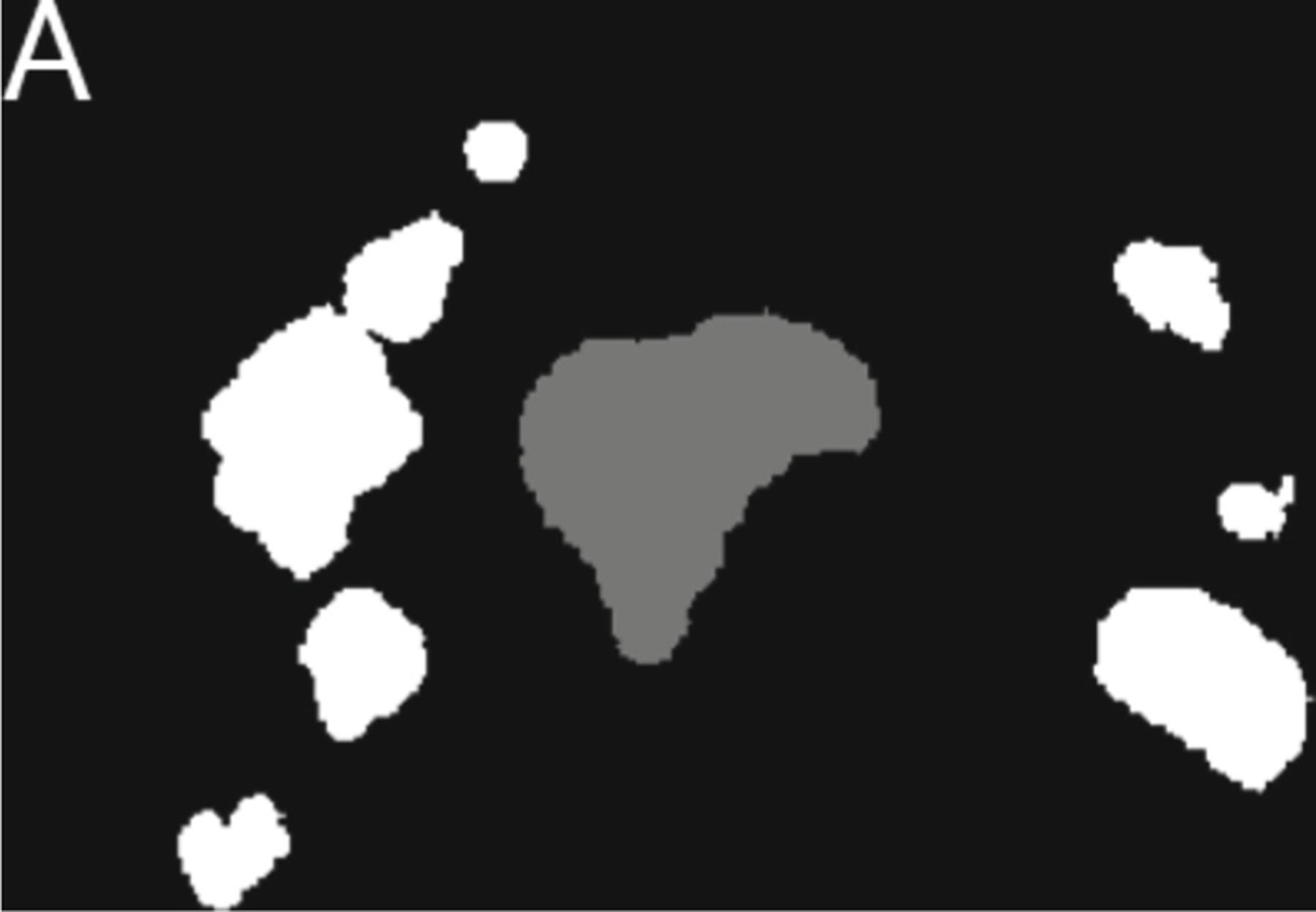
An illustration of the axial registered-pre-radiotherapy mask of case number 155 which demonstrates the 1cm expansion around all ground truth GTVp and GTVn labels. This processed mask was used in the post-processing stage of inference for Task 2.

After ensembling was conducted to generate GTVp and GTVn preliminary masks, post-processing for both Task 1 and Task 2 was performed. Post-processing involved filtering out predicted instance-lesions based on volume thresholds to minimize false positives. Specifically, for Task 1, GTVp instance-lesion predictions smaller than 200 mm^3^ and GTVn instance-lesion predictions under 60 mm^3^ were excluded. In Task 2, slightly lower thresholds were applied, with GTVp instance-lesion predictions under 175 mm^3^ and GTVn instance-lesion predictions below 50 mm^3^ being removed. These volume cutoffs were clinically determined after analyzing GTVp and GTVn volumes on a per-lesion basis across the entire 150-case dataset as described in Sect. 3.1. Additionally, for Task 2, a 1 cm expansion was applied to the registered pre-radiotherapy ground truth mask to accommodate expected GTVp and GTVn boundary extremes as shown in Fig. 3.

Predicted voxel labels for the mid-radiotherapy GTVp and GTVn outside of this expanded region were discarded, as they were presumed to be false positives. This post-processing step was crucial in enhancing prediction accuracy and ensuring that the GTVp and GTVn delineations remained clinically relevant for both tasks. Additionally, if there were any instances where a GTVp and a GTVn are directly touching, then post-processing determined whether a larger and separate GTVp lesion existed. If there was another GTVp instance-lesion, then the touching GTVp and GTVn was all converted to GTVn. If there was no other GTVp instance-lesion, then the touching GTVp and GTVn was all converted to GTVp.

### 2.5 Final Testing Evaluation

The HNTS-MRG 24 challenge final testing phase metrics included the DSCagg for GTVp and GTVn on the organizer’s hidden testing set. DSCagg has historically been used for head and neck tumor and node automated segmentation challenges given its ability to account for multiple lesions [15, 16]. Additionally, the mean DSCagg was computed based on the average of the GTVp DSCagg and GTVn DSCagg. Equation 1 shows the formula for computation of the DSCagg, where N is the total number of test images, $y_{\left\{i,k\right\}}$ is the ground truth mask for either GTVp or GTVn for voxel k of image i, and ${\hat{y}}_{\left\{i,k\right\}}$ is the model’s prediction mask [11, 15–17].


(1) $$DSC_{agg}=\frac{2\sum_{i}^{N}\sum_{k}\left({\hat{y}}_{\left\{i,k\right\}}\;\cdot \;y_{\left\{i,k\right\}}\right)}{\sum_{i}^{N}\sum_{k}\left({\hat{y}}_{\left\{i,k\right\}}\;+\;y_{\left\{i,k\right\}}\right)}$$

All metrics were computed automatically on the Grand Challenge host website using the HNTS-MRG 24 organizers evaluation metric code (https://hntsmrg24.grand-challenge.org/).

## 3 Results

### 3.1 Ground Truth Challenge Dataset Results

Analysis of the complete ground truth HNTS-MRG 24 dataset demonstrated that 12 GTVp instances in the registered pre-RT images had a volume under 16 mm^3^ among cases 3, 60, 110, 125, 125, 125, 161, 164, 169, 169, 179, 193. The next smallest GTVp instance had a volume of 192 mm^3^ for case 34. Analysis demonstrated that 13 GTVn instances in the registered-pre-radiotherapy images had a volume under 40 mm^3^. The next smallest GTVn instance had a volume of 98 mm^3^ for case 184. Analysis demonstrated that only 1 mid-radiotherapy GTVp instance had a volume of 49 mm^3^ for case 149 and the next smallest mid-radiotherapy GTVp instance had a volume of 231.5 mm^3^ for case 155. Analysis demonstrated that only 1 mid-radiotherapy GTVn instance had a volume of 0.66 mm^3^ for case 191 and the next smallest mid-radiotherapy GTVn instance had a volume of 64.5 mm^3^ for case 155. Note that all of the instance GTVp and GTVn volume calculations were performed on a lesion-wise level with 26-connected component analysis to determine distinct instance lesions. These findings suggest that post-processing thresholds should be used to remove potential false-positive instance-lesions.

Further training set analysis compared the ground truth registered-pre-radiotherapy tumor and node masks compared to the ground truth mid-radiotherapy GTVp and GTVn masks. Analysis demonstrated a lesion-wise aggregated DSCagg of 0.448 for GTVp and 0.725 for GTVn ground truth comparisons amongst the registered-pre-radiotherapy and the mid-radiotherapy masks. Further ground truth lesion-wise DSCagg comparisons were made of the registered-pre-radiotherapy ground truth masks when radially shrunken by 1–5 mm compared to the mid-radiotherapy ground truth masks. These values are shown in Table 3. These findings suggest that a simple radial reduction method from the registered-pre-radiotherapy mask could be utilized as an algorithm by itself, although this would be non-patient and non-adaptive-image specific.

### 3.2 Final Testing Phase Results

The Task 1 average cross-validation global (patient level or traditional volumetric) Dice scores at the best epochs were 0.841, 0.860, and 0.851 for GTVp, GTVn, and overall accuracy, respectively. These values are the averages of the fold level values shown in Table 1. The submitted weighted majority voting ensembled model for Task 1 achieved testing phase DSCagg scores of 0.82, 0.72, and 0.77 for GTVp, GTVn, and overall accuracy, respectively.

The Task 2 average cross-validation global (patient level or traditional volumetric) Dice scores at the best epochs were 0.507, 0.727, and 0.617 for GTVp, GTVn, and overall accuracy, respectively. These values are the averages of the fold level values shown in Table 2. The submitted majority voting ensembled model for Task 2 achieved testing phase DSCagg scores of 0.49, 0.81, and 0.65 for GTVp, GTVn, and overall accuracy, respectively.

For both Task 1 and Task 2, given that there was no left out challenge cases for independent model testing evaluation of the DSCagg, it was not possible to perform comparison of local independent testing vs the provided testing phase performance for DSCagg since the loss used in the model’s validation evaluation was the global (patient level or traditional volumetric) Dice score. However, it is hypothesized that the interim best epoch validation traditional DSC scores are higher than the testing phase DSCagg given that they report the best epoch validation DSC across the entire model training and there may not have been significant DSCagg penalties using the traditional DSC for smaller missed lesions.

The final testing phase metrics were provided by the challenge organizers. No other final test phase metrics are available at this time.

## 4 Discussion

This study utilized a SegResNet deep learning architecture as part of the MONAI and Auto3DSeg frameworks to evaluate and infer OPC primary tumor and nodal disease on T2 MRI in the pre-radiotherapy and mid-radiotherapy settings as part of the HNTS-MRG 24 challenge (hntsmrg24.grand-challenge.org/overview/) [9–11].

The achieved test set dice scores of 0.72 for GTVp and 0.82 for GTVn in Task 1, indicate robust performance in pre-treatment segmentation. These results align with recent advancements in deep learning-based segmentation, where ensemble approaches have consistently outperformed single-model predictions by mitigating individual model biases and enhancing overall accuracy [18, 19].

In Task 2, the DSCagg of 0.49 for GTVp and 0.81 for GTVn reveal a dichotomy in segmentation performance between GTVp and GTVn at the mid-radiotherapy stage. The superior performance in GTVn segmentation can be attributed to the distinct anatomical features and consistent response of lymph nodes to radiotherapy, which may present as more homogeneous changes compared to primary OPC tumors [20, 21]. Conversely, the lower dice score for GTVp in mid-radiotherapy highlights the inherent challenges in accurately capturing dynamic tumor responses, such as heterogeneity in tissue density and irregular shrinkage patterns [22]. This discrepancy underscores the necessity for further refinement of segmentation algorithms to better accommodate the complex morphological transformations of primary tumors during treatment. In future iterations of this study, implementing a larger radial expansion during the pre-processing phase should be considered to encompass a greater portion of the surrounding anatomical structures. This approach may enhance the model’s contextual understanding and improve segmentation accuracy. However, in the current study, the application of bounding boxes to generate smaller image regions was essential to address the limitations imposed by training time constraints associated with processing larger images.

When comparing this study’s DSCagg performance for GTVp and GTVn based on MRI compared to prior head and neck segmentation challenge performance based on PET/CT imaging, notable differences are appreciated. Salahuddin et al. reports an DSCagg of 0.774 and 0.760 on the test set for GTVp and GTVn, respectively [23]. Additionally, Chu et al. utilized a Swin-UNETR CNN architecture for head and neck tumor automated segmentation based on PET/CT and reported a DSCagg of 0.642, 0.670, and 0.656 for GTVp, GTVn, and overall accuracy, respectively [17]. This study’s Task 1 performance DSCagg of 0.82 for GTVn was higher than the prior work on PET/CT automated segmentation, but the DSCagg of 0.72 was similar to the prior work on PET/CT automated segmentation [17, 22]. Additionally, this challenge’s Task 2 performance DSCagg of 0.81 for GTVn was higher than the prior work on PET/CT automated segmentation, but the DSCagg of 0.49 was notably lower than the prior work on PET/CT automated segmentation [17, 22]. Further investigation should evaluate the reasoning for differing performance in GTVn and GTVp automated segmentation on PET/CT based imaging compared to pre-radiotherapy and mid-radiotherapy MR imaging.

The lesion-wise instance average Dice scores presented in Table 3 offer valuable insights into the prognostication of tumor and nodal size reductions during radiotherapy for head and neck cancers. Specifically, the GTVp Dice score peaks at a 2 mm radial reduction (0.490), suggesting that a 2 mm decrease in GTVp size from pre-radiotherapy to mid-radiotherapy aligns most closely with the ground truth segmentation. This indicates that, on average, GTVp may undergo a significant reduction of approximately 2 mm during the early phases of radiotherapy, providing a measurable benchmark for treatment efficacy. Conversely, the GTVn Dice score is highest at a 1 mm reduction (0.847), implying that GTVn structures exhibit a more modest decrease in size, with a 1 mm reduction being the most accurate reflection of their true anatomical changes during treatment. These differential reduction patterns between tumors and nodes underscore the heterogeneous nature of tissue responses to radiotherapy. Interestingly, this study’s Task 2 model’s test set mid-radiotherapy GTVn DSCagg of 0.81 was lower than the simple registered-pre-radiotherapy mask reduction amplitude of 1 mm, which had a Dice score of 0.847 averaged across the entire training dataset when compared to the respective case’s mid-radiotherapy GTVn mask. In spirit of the challenge, this study did not utilize the simple 1 mm radial mask reduction, as this should not be a method used in the clinic for adaptive radiotherapy planning as it is not patient specific or on-treatment image specific. Similarly, the Task 2 model’s test set mid-radiotherapy GTVp DSCagg of 0.490 was identical to the simple registered-pre-radiotherapy mask reduction amplitude of 2 mm across the whole training set. Further analysis of the testing set using these simple radial reductions algorithms would be interesting for comparison to this challenge’s participant’s deep learning models. Further studies should also evaluate the inter-observer variability between annotators for each case in the challenge training dataset to determine whether human-human Dice is similar, worse, or better, than the challenge’s participant’s automated segmentation models when compared to the STAPLE consensus ground truths.

Utilizing the Dice scores associated with radial shrinkage of registered-pre-radiotherapy masks as prognostic markers could allow clinicians to better predict treatment outcomes, tailor radiotherapy plans, and adjust therapeutic strategies in real-time to enhance precision and effectiveness using adaptive radiotherapy planning [8, 24, 25]. The adaptive radiotherapy automated segmentation models could use the radial reduction masks for GTVp and GTVn as additional data when inferring the real time GTVp and GTVn target volume delineation. However, it is essential to consider individual patient variability and the potential influence of factors such as tumor biology and treatment modalities, which may affect the generalizability of these findings. Future studies should aim to validate these prognostic thresholds in larger, diverse cohorts and explore the underlying mechanisms driving the differential responses of tumors and nodes to radiotherapy.

Notably, the DSCagg in the HNTS-MRG 24 challenge is used similarly to the modified lesion-wise DSC used in the 2024 BraTS meningioma radiotherapy automated segmentation challenge as shown in Eq. 2, and it is similar to the lesion-wise DSC used in the BraTS preoperative meningioma, glioma, metastasis, sub-saharan Africa glioma, and pediatric brain tumor automated segmentation challenges as shown in Eq. 3 [26–31].


(2) $$Lesion\;wise\;DSC=\frac{\sum_{i}^{L}\text{Dice}\left({\text{I}}_{i}\right)}{\text{TP}+\text{FN}+\text{FP}}$$

(3) $$Modified\;lesion\;wise\;DSC=\frac{\sum_{i}^{L}\text{Dice}\left({\text{I}}_{i}\right)}{\text{TP}+\text{FN}}$$

In Eqs. 2 and 3, L is the number of ground truth lesions. Predicted true positive (TP) + false negative (FN) lesions is equal to L. A predicted lesion is counted as a TP if at least 1 predicted voxel overlaps with the respective ground truth’s respective region of interest mask. A lesion is counted as a FN if the model does not predict any voxels within the ground truth’s respective region of interest mask. A predicted lesion is counted as a false positive (FP) if the model predicts a distinct lesion that does not overlap with any ground truth lesions’ voxels [26].

The modified lesion-wise DSC used in the 2024 BraTS meningioma radiotherapy automated segmentation challenge does not penalize for false positive instance lesion predictions, as radiotherapy planning scans are typically not used for diagnostics, but rather for the contouring workflow [28]. Additional predicted instance lesions on radio-therapy images can easily be removed during the contouring workflow using a Boolean tool or other similar tools and would not significantly detract from the amount of time needed to contour, and therefore false positives can be considered to not need to be penalized in performance metrics. Additionally, localizing and contouring additional lesions that were not automatically contoured could theoretically take excess time and therefore false negatives should still be considered in performance metrics. Future automated segmentation studies and challenges using radiotherapy planning images, should consider not penalizing false positive instance lesions as these can be removed relatively easily during the radiotherapy planning process.

Unfortunately, due to participant time limitations, only the SegResNet network was used as part of the Auto3DSeg architecture, whereas previous studies have utilized each of the SegResNet, DiNTS, and Swin-UNETR CNN and transformer-based components with sliding window inferential ensembling [9, 10, 32]. If additional time was available, then additional models using the DiNTS and Swin-UNETR CNN architectures with an iSTAPLE ensembling technique would have been utilized and future studies should consider using this method.

Future studies should also consider using a larger GPU than this study’s RTX 2070 to allow for more complex model structures, as well as more consistent and thorough training. By increasing the number of parameters, increasing the number of epochs, and utilizing additional data augmentation, a greater model accuracy will likely be achieved for both Task 1 and Task 2.

## 5 Conclusion

This study developed and validated SegResNet deep learning models for the automated segmentation of GTVp and GTVn in OPC using pre-treatment and mid-treatment T2 MRI using the Auto3DSeg and MONAI frameworks [9, 10]. By integrating model ensembling with specific pre- and post-processing techniques, our models testing DSCagg scores of 0.72 and 0.82 for GTVn and GTVp in pre-treatment MRI (Task 1), and 0.81 and 0.49 for GTVn and GTVp in mid-treatment MRI (Task 2). These results highlight the effectiveness of deep learning-based automated segmentation in enhancing radiation oncology workflows. Future studies will focus on improving mid-treatment GTVp and GTVn segmentation performance and exploring the clinical implications of automated tumor delineation in adaptive radiotherapy.

## Figures and Tables

**Table 1.** Task 1 fold-wise overall accuracy for the fine-tuned SegResNet models. The overall accuracy (Best Validation Dice or Final Validation Dice) was computed as the average of the GTVp and GTVn global (patient level or traditional volumetric) Dice scores. The models were trained over various epochs with cross-validation. Note that fold 0 used the exact same validation cases as the pre-trained model, whereas folds 1–5 each had 12.5% of their validation cases within the pre-trained model’s training sets; therefore explaining the artificially high Best Validation Dice.

Fold	Epochs Completed	Best Validation Dice (GTVp, GTVn)	Best Epoch	Final Validation Dice (GTVp, GTVn)	Model Weight for Ensembling
0	556	0.7292 (0.694, 0.765)	59	0.7004 (0.664, 0.737)	0.2
1	335	0.8766 (0.881, 0.872)	9	0.8522 (0.847, 0.858)	0.1375
2	556	0.8814 (0.873, 0.889)	9	0.8748 (0.858, 0.892)	0.175
3	556	0.8651 (0.868, 0.862)	19	0.8593 (0.848, 0.871)	0.175
4	556	0.8785 (0.863, 0.894)	9	0.8612 (0.850, 0.872)	0.175
5	465	0.8719 (0.866, 0.878)	9	0.853 (0.836, 0.870)	0.1375

**Table 2.** Task 2 fold-wise overall accuracy for the SegResNet models. The overall accuracy (Best Validation Dice or Final Validation Dice) was computed as the average of the GTVp and GTVn global (patient level or traditional volumetric) Dice scores. The models were trained over 534 planned epochs with 5-fold cross-validation.

Fold	Epochs Completed	Best Validation Dice (GTVp, GTVn)	Best Epoch	Final Validation Dice (GTVp, GTVn)	Model Weight for Ensembling
0	534	0.5967 (0.530, 0.663)	456	0. 5859 (0.513, 0.659)	0.2
1	534	0.6223 (0.559, 0.686)	466	0.6160 (0.546, 0.686)	0.2
2	534	0.6578 (0.521, 0.795)	340	0.6541 (0.510, 0.798)	0.2
3	534	0.5879 (0.433, 0.743)	300	0.5620 (0.368, 0.756)	0.2
4	534	0.6341 (0.492, 0.776)	332	0.6287 (0.488, 0.769)	0.2

**Table 3.** These data show the ground truth Dice comparisons of the registered-pre-radiotherapy ground truth masks when radially shrunken by 0–5 mm compared to the mid-radiotherapy ground truth masks.

Registered-pre-radiotherapy mask reduction amplitude	GTVp Dice	GTVn Dice
0 mm	0.448	0.725
1 mm	0.135	0.847
2 mm	0.490	0.651
3 mm	0.441	0.518
4 mm	0.370	0.412
5 mm	0.331	0.313
